# Closed Genome Sequences of Clinical Listeria monocytogenes PCR Serogroup IVb Isolates Associated with Two Recent Large Listeriosis Outbreaks in Germany

**DOI:** 10.1128/MRA.01434-20

**Published:** 2021-02-04

**Authors:** Sven Halbedel, Martin A. Fischer, Andrea Thürmer, Antje Flieger

**Affiliations:** a FG11 Division of Enteropathogenic Bacteria and Legionella, Robert Koch Institute, Wernigerode, Germany; b MF2 Genome Sequencing, Robert Koch Institute, Berlin, Germany; University of Rochester School of Medicine and Dentistry

## Abstract

Here, we report the closed genome sequences of two representative Listeria monocytogenes strains belonging to PCR serogroup IVb, which are related to two large outbreaks of human listeriosis that affected Germany in 2015 (Eta1) and 2018 to 2019 (Epsilon1a).

## ANNOUNCEMENT

The Gram-positive bacterium Listeria monocytogenes is widespread in the environment and frequently contaminates various food products. Consumption of contaminated food can lead to listeriosis, which is associated with high lethality rates (1).

The German molecular surveillance program has uncovered several listeriosis outbreaks in the recent past, based on Illumina short-read sequencing and 1,701-locus core genome multilocus sequence typing (cgMLST) (2, 3). Among these outbreaks were two listeriosis clusters caused by two different PCR serogroup IVb clones belonging to multilocus sequence typing (MLST) sequence type 6 (ST6) and ST2. Further cgMLST analyses assigned complex type 4465 (CT4465) and CT7353 to the ST6 isolates (referred to as Epsilon1a) and CT1114 to the ST2 isolates (Eta1) (4, 5).

Here, we report the closed genome sequences of one representative isolate of each of these two outbreak clusters (Table 1). Isolates 15-01121 (Eta1) and 18-04540 (Epsilon1a) were grown in brain heart infusion (BHI) broth overnight at 37°C, and genomic DNA was extracted using the phenol-chloroform method (6). DNA quality and concentration were determined using a NanoDrop spectrophotometer (Thermo Fisher Scientific, Waltham, MA, USA).

**TABLE 1:** Key characteristics of the genomes sequenced in this report

Parameter	Data for isolate:	
	18-04540	15-01121
Outbreak	Epsilon1a	Eta1
Source	Clinical isolate	Clinical isolate
Yr of isolation	2018	2015
GenBank accession no.	CP063383	CP063382
ENA accession no.	SAMEA5041142	SAMEA104485140
Sequencing method	MinION	PacBio
Accession no. for long-read raw data	ERX4581157	ERX4581155
Accession no. for short-read raw data	ERX2860946, ERX4866683	ERX2313138
Genome size (bp)	2,992,699	2,995,602
GC content (%)	37.98	37.98
No. of protein-coding genes	2,923	2,897
No. of rRNA operons	6	6
No. of tRNA genes	67	67
Pathogenicity island(s)	LIPI-1, LIPI-3	LIPI-1
Plasmid (GenBank accession no.)	pLMST6 (CP063384)	None
PCR serogroup	IVb	IVb
ST^*a*^	ST6	ST2
CT^*b*^	CT4465	CT1114
Outbreak yr(s)	2018–2019	2015
Reference no.	5	4

a ST according to 7-locus MLST (13).

b CT according to 1,701-locus cgMLST (2).

A SMRTbell template library was prepared from chromosomal DNA of isolate 15-01121 according to the instructions from Pacific Biosciences (Menlo Park, CA, USA), following the procedure and checklist for 20-kb template preparation using the BluePippin size selection system. The PacBio long-read library was sequenced with one single-molecule real-time (SMRT) cell on a PacBio RS system (Pacific Biosciences) using the 240-min movie run mode according to the manufacturer’s standard protocol with components from the DNA sequencing kit 4.0 v2 by GATC Biotech (Konstanz, Germany) generating 64,270 raw reads. SMRT Portal v2.3.0 software (Pacific Biosciences) was used for read filtering and adapter trimming with default parameters. The 22,116 high-quality long reads obtained (*N*_50_, 9,941 bp) were used for further genome assembly. The same isolate had been sequenced previously using 2 × 300-bp paired-end Illumina sequencing chemistry, generating 1,256,850 reads (ENA accession number ERX2313138) (4).

The library for isolate 18-04540 was prepared using the SQK-RKB004 kit and sequenced on a MinION instrument in combination with a 1D flow cell (Oxford Nanopore Technologies, Oxford, UK), generating 698,275 long reads. After quality filtering using NanoFilt (7) with default parameters, a subset of 10^5^ reads (*N*_50_, 17,981 bp) was used for genome assembly. Isolate 18-04540 was also sequenced twice on an Illumina MiSeq instrument using single-direction 1 × 150-bp chemistry, generating 1,318,886 reads in total (ENA accession numbers ERX2860946 and ERX4866683) (5).

Illumina raw reads were trimmed using Trimmomatic v0.36 (8) with standard parameters before the assembly. For isolate 18-04540, Oxford Nanopore Technologies long reads were subsampled to 25,000 sequences. Circular genome assemblies were generated using Unicycler v0.4.8 (9) with standard parameters in hybrid assembly mode using long- and short-read sequences. Correct circularization was verified by read mapping to the final assembly. Statistics for the assembled genomes were obtained using QUAST v5.0.0 (10).

The assembled genome of 15-01121 had a sequencing depth of 84-fold, a length of 2,995,602 bp, and a GC content of 37.98%. The assembled genome of 18-04540 had a sequencing depth of 59-fold, a length of 2,992,699 bp, and a GC content of 37.98%. Isolate 18-04540 contained the already described plasmid pLMST6, conferring benzalkonium chloride tolerance (11), with a length of 4,265 bp and a GC content of 36.76%. Both genomes were annotated using the NCBI Prokaryotic Genome Annotation Pipeline (PGAP) v4.13 (12).

The Epsilon1a outbreak, with 112 affected patients, was one of the largest outbreaks of invasive listeriosis seen in Europe in 25 years (5), while the Eta1 outbreak was a solely gastroenteritis outbreak affecting 163 patients (4). The genome sequences reported here might further promote the analysis of these two interesting L. monocytogenes clones.

### Data availability.

All genome sequences were deposited at NCBI or ENA, and their accession numbers are given in Table 1.
